# Supplementary material for: Using Behavior Integration to Identify Barriers and Motivators for COVID-19 Vaccination and Build a Vaccine Demand and Confidence Strategy in Southeastern Europe
Source: Vaccines (Basel). 2024 Oct 2;12(10):1131. doi: 10.3390/vaccines12101131 (PMC11511038; doi:10.3390/vaccines12101131)
Supplement: Supplementary file 1 [file vaccines-12-01131-s001.zip › Supplementary Material 1.pdf]

Supplementary Material 1. Factors and Definitions to Consider when Using Behavior Integration

Among structural factors, accessibility ensures that individuals can obtain necessary resources and services. The quality of experience with providers and supporting actors, as well as products, services, groups, companies, and institutions, also shape user engagement and satisfaction. Social factors encompass support dynamics from family, peers, and the broader community, alongside gender influences that often dictate roles and behaviors within societal norms. Internal factors including attitudes and beliefs, self-efficacy, and levels of knowledge and skills are equally significant. Each identified factor and sub-factor contribute to behavior change processes and requires analysis and strategic planning for effective program implementation.

Table 1: Factors and Definitions

**Factor**	**Definition**
**Structural**	
**Accessibility**	The primary actor's ability to practice the behavior given external constraints, including physical, economic, political, and policy.
**Experience with provider or other supporting actor**	The primary actor's perception of the experience with a person associated with a product, service, group, company, or institution, and supervisor or other supporting actor.<br><br>Note: This is only applicable if the primary actor uses a service. If the service provider (e.g., health care worker, government employee, business) is the primary actor, his/her actual competencies should be considered under "Skills."
**Experience with product, service, group, company, or institution**	The primary actor's perception of his/her experience with a product, service, group, company, or institution.
**Social**	
**Family, peer, and community support**	Proactive or passive help, encouragement, or attitudes toward a behavior by family members, peers, colleagues, and others in the community at large.
**Gender**	The social rules and expectations related to gender that influence the practice of a behavior.
**Norms**	The acceptability and standards for practice of a behavior determined by religious, cultural, workplace, and other social rules and expectations.
**Internal**	

**Attitudes and beliefs**	The primary actor's judgment, feeling, and emotion about a behavior.
**Self-efficacy**	The primary actor's confidence, agency, and conviction to practice a behavior.<br><br>Note: This factor may not be applicable in many cases beyond health.
**Knowledge**	The primary actor's information required to practice a behavior.
**Skills and capacity**	The primary actor's ability to perform a set of tasks required to practice a behavior.
