# Supplementary material for: Using Behavior Integration to Identify Barriers and Motivators for COVID-19 Vaccination and Build a Vaccine Demand and Confidence Strategy in Southeastern Europe
Source: Vaccines (Basel). 2024 Oct 2;12(10):1131. doi: 10.3390/vaccines12101131 (PMC11511038; doi:10.3390/vaccines12101131)
Supplement: Supplementary file 1 [file vaccines-12-01131-s001.zip › Supplementary Material 10.pdf]

Supplementary Material 10. CME Note-taking Tool

Learning note-taking template: CME trainers								
**Date of entry**	**Name of notetaker**	**Location (district, regional, national) - optional**	**Date of training - optional**	**Describe your overall reflections about the CME training?**<br><br>***(Describe what happened at the training in general)***	**What went well and why?**<br><br>***(Briefly describe what steps or aspects of the CME training worked well, and why)***	**(important learning question)** **How well are provider responding to different techniques in the CME training?**<br><br>**Are some techniques working better than others, and if they are, why?**<br><br>**Techniques: role plays, case studies, self-assessments, games**	**What barriers or challenges were encountered during the training?**	**How can we make the CME training better? Can you give us some examples?**

Learning note-taking template for CME trainees							
**Date of entry**	**Name of notetaker**	**Location (district, regional, national)**	**Describe your overall reflections about the CME training?**<br><br>***(Describe what happened at the training in general)***	**What did you like/enjoy about the training, and why?**	**(important learning question) Which techniques in the CME training did you find the most helpful, and why? Which ones did you find least helpful, and why?**<br><br>**Techniques: role plays, case studies, self-assessments, games**	**(important learning question) Did the CME training provide you with sufficient understanding to confidently recommend the COVID-19 vaccine to your patients?**	**How can we make the CME training better? Can you give us some examples?**
