# Supplementary material for: Using Behavior Integration to Identify Barriers and Motivators for COVID-19 Vaccination and Build a Vaccine Demand and Confidence Strategy in Southeastern Europe
Source: Vaccines (Basel). 2024 Oct 2;12(10):1131. doi: 10.3390/vaccines12101131 (PMC11511038; doi:10.3390/vaccines12101131)
Supplement: Supplementary file 1 [file vaccines-12-01131-s001.zip › Supplementary Material 11.pdf]

Supplementary Material 11. CE Note-taking Tool

**Learning note-taking template: CE facilitators**								
**Date of entry**	**Name of notetaker**	**Location (district, regional, national) - optional**	**Date of training (optional)**	**Describe your overall reflections about the CE event?**<br><br>***(Describe what happened at the training in general)***	**What went well and why?**<br><br>***(Briefly describe what steps or aspects of the CE activity worked well, and why)***	**(important learning question) How well are participants responding to the different activities in the CE event?**<br><br>**Are some activities working better than others, and if they are, why?**	**What barriers or challenges were encountered during the CE activity?**	**How can we make the CE activity better? Can you give us some examples?**

Learning note-taking template for CE participants							
Date of entry	Name of notetaker	Location (district, regional, national)	Share your general reflections about the CE event?	What aspects of the CE event did you like, and why?	(important learning question) Which activities in the CE event did you find the most helpful for learning about COVID-19 vaccines and why? Which activities did you find least helpful, and why?<br><br>Activities: role plays, self-assessments, games	(important learning question) Did the CE event provide you with enough information to decide to get the COVID-19 vaccine? Why or why not?	How can we make the CE event better? Can you give us some examples?
