# Supplementary material for: Using Behavior Integration to Identify Barriers and Motivators for COVID-19 Vaccination and Build a Vaccine Demand and Confidence Strategy in Southeastern Europe
Source: Vaccines (Basel). 2024 Oct 2;12(10):1131. doi: 10.3390/vaccines12101131 (PMC11511038; doi:10.3390/vaccines12101131)
Supplement: Supplementary file 1 [file vaccines-12-01131-s001.zip › Supplementary Material 2.pdf]

Supplementary Material 2. Supporting Actors to Consider when Using Behavior Integration

Table 1: Supporting Actors and Definitions

Potential Actor, by level	Definition
**Institutional**	
**Policymaker**	Designs and implements policies.
**Manager**	Supervises others, including the primary actor or person providing a service to the primary actor.
**Logistics personnel**	Manage product or commodity supply chains at all levels.
**Provider**	Directly provides services to a client or customer.
**Employer**	Person or organizations that employ people.
**Community**	
**Community leader**	Has influence over and represents a community.
**Religious leader**	Has authority and influence within a religion.
**Teacher**	Teaches others, usually in a school setting.
**Household**	
**Family member**	Immediate or extended relative such as parent, grandparent, aunt, uncle, and sibling.
**Male partner**	Spouse, boyfriend, and other male companion.
