# Supplementary material for: Using Behavior Integration to Identify Barriers and Motivators for COVID-19 Vaccination and Build a Vaccine Demand and Confidence Strategy in Southeastern Europe
Source: Vaccines (Basel). 2024 Oct 2;12(10):1131. doi: 10.3390/vaccines12101131 (PMC11511038; doi:10.3390/vaccines12101131)
Supplement: Supplementary file 1 [file vaccines-12-01131-s001.zip › Supplementary Material 3.pdf]

Supplementary Material 3. In-depth Stakeholder Interview Guide Conducted as a Part of the Formative Assessment

Interviewer: Document the following information about the respondent

Name of interviewer:

Respondent code:

Study site:

Date of interview:

Start time of interview:

End time of interview:

Audio recording file name:

These questions are to be used as a guide for initiating and stimulating discussions during the in-depth interviews. Please note per the interviewer's discretion, some questions may be skipped or prioritized depending upon the type of stakeholder being interviewed (i.e., national/ county), the area of work, and the time available for the interview.

INTERVIEW GUIDE

A. Background

1. Can you tell me a little about yourself?

    Probes: Background, experience, position, role in COVID-19 response, how long you have been in this position

B. COVID-19 vaccinations trends and their barriers and facilitators

2. What have been [country's] experience with COVID-19 vaccines?

3. What have been the major barriers to COVID-19 vaccinations? Why?

    Probes: mis/disinformation; inadequate access to health services; language barriers; trust in health systems/providers; individual beliefs (e.g., fear of vaccines); mistrust of pharma; questions about vaccine safety/effectiveness

    [Interviewer: Explore whether any of the barriers differ by county, population group or any other factors, such as age, sex, etc.]

4. What have been the motivators to COVID-19 vaccinations? Why?

    Probes: collective immunity/sense of responsibility toward others; internal motivation (e.g., protect oneself from disease); financial incentives (e.g., shopping vouchers, concert tickets, travel vouchers); mandates by employer/government

[Interviewer: Explore whether any of the facilitators differ by county, population group or any other factors, such as age, sex, etc.]

5. In your view, which groups have been most/least willing to get vaccinated? Why?

a. Are any of these population groups viewed as high-priority by gov't (national or county)? Why?

Probes: Elderly (60 years and older), youth, rural, less educated, health workers, etc.

[Interviewer: Explore whether the unvaccinated population groups differ by county and by other additional factors]

6. Have there been any strategies/programs to increase COVID-19 vaccine uptake?

a. Can you tell me more about these targets? Who do these strategies target?

b. To what extent, have these strategies been successful? What factors are needed for these strategies to be implemented?

c. Who has been responsible for implementing these strategies?

d. Where can I get more information about these strategies?

C. Actors in the COVID-19 vaccination landscape

7. Who are the various actors involved with COVID-19 vaccine roll out and uptake in [country] at the national and subnational levels?

a. Who is responsible for overseeing the overall Covid-19 vaccination roll out?

b. Who is responsible for collating and reviewing vaccine coverage data for decision-making?

c. Who is responsible for designing programs and strategies to reach unvaccinated populations groups?

d. Who is involved with communications and advocacy efforts relating to COVID-19 vaccines?

8. More broadly, what are the actors in support/opposition to COVID-19 vaccines? Why do they feel that way?

[Interviewer: Ask whether any of the actors are from non-government/community-based organizations, or development partners, such as WHO/UNICEF]

D. Stakeholder specific section: Covid-19 vaccine data use among public health stakeholders

[Note: this section will be tailored to different stakeholders. The topics listed below are examples of questions that will be explored among public health decision-makers, such as individuals from the national/regional institutes of public health, health associations, and ministry of health]

9. How are you currently making programmatic decisions around COVID-19 vaccines?

a. What types of factors drive your decision-making?

b. Specifically, how is data being used to inform programmatic decisions around COVID-19 vaccinations, what populations to target, etc.?

10. Who are the actors involved with using the COVID-19 vaccination data most in [organization]? Can you give an example of how these data are used?

11. How does [organization] engage other external stakeholders to discuss these data? In what avenues are these data discussed, and what decisions are made on their basis?

E. Learning exchange and avenues

12. What are existing mechanisms (if any) for promoting data use or learning exchanges among immunization stakeholders in [country]?

a. How are these structured? How frequently do you meet? What do you discuss?

b. Are these avenues effective? Why or why not?

F. Closing

13. Based on our discussion, are there any other individuals or organizations who are important stakeholders in the COVID-19 vaccination landscape who you would recommend us speaking with?
