# Supplementary material for: Using Behavior Integration to Identify Barriers and Motivators for COVID-19 Vaccination and Build a Vaccine Demand and Confidence Strategy in Southeastern Europe
Source: Vaccines (Basel). 2024 Oct 2;12(10):1131. doi: 10.3390/vaccines12101131 (PMC11511038; doi:10.3390/vaccines12101131)
Supplement: Supplementary file 1 [file vaccines-12-01131-s001.zip › Supplementary Material 4.pdf]

***Supplementary Material 4***. Quantitative Online Survey (PREMISE Survey)

| S/N | Segmentation category (i.e., factor to help group respondents) | Question | Question type | Response options | Skip logic |
|---|---|---|---|---|---|
| 1 | Language | What language(s) do you speak? | Multiple choice (Select many) | English = 0<br>Serbian = 1<br>Macedonian = 2<br>Moldovan = 3<br>Russian = 4<br>Romanian = 5 | |
| 2 | Religion | What religion do you identify with? | Multiple choice | Orthodox<br>Protestant (Evangelical,<br>Baptist, Jehovah witness)<br>Jewish<br>Islam<br>Other | |
| 3 | Ethnicity | What ethnicity do you identify with? | Multiple choice | Serbian<br>Moldovan<br>Ukrainian<br>Russian<br>Gagauzan<br>Romain<br>Bulgarian<br>Other | |
| 4 | Role as a health worker | Are you a health professional involved in vaccinating or counseling patients about the COVID-19 vaccine | Y/N | No =0<br>Yes = 1 | |
| 5 | Priority behaviors to influence | Have you received 1 or more doses of COVID-19 vaccines? | Multiple choice | No =0<br>Yes = 1 | If response is "Yes", skip to 6 |

| | | | | | |
|---|---|---|---|---|---|
| 6 | Intention to get vaccinated | Do you want to get a COVID-19 vaccine? Would you say… | Likert | No, you do not want to = 0<br>Yes, you do want to = 1<br>You are not sure = 2 | [For those who responded "No" Q4 will answer this question] |
| 7 | Fully vs partially vaccinated against COVID-19 vaccine | Would you say you are fully or partially vaccinated against COVID-19 vaccine?<br>Fully vaccinated means…<br>Partially vaccinated means… | Multiple choice | Partially vaccinated = 0<br>Fully vaccinated = 1 | |
| 8 | Confidence in health workers | How much do you trust the health workers who gave you/would give you a COVID-19 vaccine? Would you say… | Likert | Not at all = 0<br>Very little = 1<br>Somewhat = 2<br>To a great extent =3 | |
| 9 | Social: Peer norms | Have most of the adults you know gotten a COVID-19 vaccine? | Yes/No | No =0<br>Yes =1 | |
| 10 | Social: Workplace norms | Have most of the people you work with gotten a COVID-19 vaccine? | Yes/No | No =0<br>Yes =1<br>Not currently working = 2 | |
| | Social: Family, Community and Religious leader norms | Who do you think wanted you to, or would like you to, get a COVID-19 vaccine? | Multiple choice question<br>(select many) | Close family and friends = 0<br>Religious leaders = 1<br>Community leaders = 2 | |
| 11 | Social: Health worker recommendation | Has a health worker recommended you get a COVID-19 vaccine? | Yes/No | No =0<br>Yes =1 | |
| 12 | Access:<br>Know where to get the vaccination | Do you/did you know where to go to get a COVID-19 vaccine for yourself? | Yes/No | No =0<br>Yes =1 | |
| 13 | Access: Ease of access | How easy is it to get a COVID-19 vaccine for yourself? Would you say… | Likert | Not at all easy = 0<br>A little easy = 1<br>Moderately easy = 2<br>Very easy =3 | |

| | | | | | |
|---|---|---|---|---|---|
| | *Potential alternative to question above* | Which of the following issues do you think about when considering the affordability of getting the COVID-19 vaccination? | Multiple choice<br><br>(Select many) | Payment to clinic = 1<br><br>Transportation to get to the clinic = 2<br><br>Time away from work = 3<br><br>Other = 4 | |
| 14 | Access:<br><br>Reasons for low ease of access | What are the barriers you have faced in getting a COVID-19 vaccine? What makes it hard for you to get a COVID-19 vaccine? Would you say… | Multiple choice (select all that apply) | Nothing, it's not hard =0<br><br>I am not eligible for COVID-19 vaccines =1<br><br>Making an appointment is hard (e.g., technical, website, phone difficulties) =2<br><br>The vaccination site is hard to get to=3<br><br>No vaccine appointments=4<br><br>Unable to provide required documents=5<br><br>Waiting time takes too long=6<br><br>I am unable to leave work duties/school/child care=7<br><br>Information not available in native language=8<br><br>Couldn't get the type of vaccine wanted=9<br><br>Sometimes people are turned away without vaccination=10 | |
| 15 | Internal:<br><br>Attitudes and Beliefs | What are the reasons for getting vaccinated? Would you say… | Multiple choice (select all that apply) | COVID-19 vaccines are effective = 0<br><br>Getting COVID-19 vaccines is a good way to prevent/treat disease =1<br><br>COVID-19 vaccines are important for health = 2<br><br>Receiving COVID-19 vaccines is important for the health of the community =3<br><br>Other _____ | |
| 16 | Internal:<br><br>Attitudes and Beliefs | Do you think COVID-19 continues to remain a threat to the broader community you live in? Would you say… | Likert | Not at all = 0<br><br>Very little = 1<br><br>Somewhat = 2<br><br>To a great extent =3 | |

| | | | | | |
|---|---|---|---|---|---|
| 17 | Internal:<br>Attitudes and Beliefs | Do you think that newly developed vaccines, like those for COVID-19, carry more risks than established/routine vaccines? Would you say… | Likert | Not at all = 0<br>Very little = 1<br>Somewhat = 2<br>To a great extent =3 | |
| 18 | Internal:<br>Attitudes and Beliefs | How concerned are you about serious adverse events of COVID-19 vaccines? | Likert | Not at all = 0<br>Very little = 1<br>Somewhat = 2<br>To a great extent =3 | |
| 19 | Internal:<br>Attitudes and Beliefs | Do you do whatever your health provider recommends about vaccines? | Likert | Not at all = 0<br>Very little = 1<br>Somewhat = 2<br>To a great extent =3 | |
| 20 | Service experience:<br>Technical competencies of service providers | To what extent do you feel health providers you have interacted with make you feel comfortable at your local health facilities? | Likert | Very uncomfortable = 0<br>Uncomfortable = 1<br>Comfortable = 2<br>Very comfortable = 3 | |
| 21 | Service experience:<br>Technical competencies of service providers | To what extent do you feel you understand the information your health provider is sharing with you? | Likert | Not at all = 0<br>Sometimes =1<br>Often =2<br>Always =3 | |
| 22 | Service experience:<br>Satisfaction | How satisfied are you with COVID-19 vaccination services? Would you say… | Likert | Not at all satisfied = 0<br>A little satisfied = 1<br>Moderately satisfied = 2<br>Very satisfied = 3 | |

| | | | | | |
|---|---|---|---|---|---|
| 23 | Service experience:<br>Quality | What is not satisfactory about the COVID-19 vaccination services? Would you say… | Multiple choice (select all that apply) | Nothing, you are satisfied = 0<br>Vaccine is not available= 1<br>The vaccination site does not open on time=2<br>Waiting times are long=3<br>The vaccination site is not clean=4<br>Staff are poorly trained=5<br>Staff are not respectful=6<br>Staff do not spend enough time with people=7<br>Other____=8 | |
| 24 | Information sources: Trusted | Who do you trust in your community to provide you with information about COVID-19? Please rank the following from high to low trust. | Likert | [Ranking question]<br>Local health workers<br>Scientists and health experts<br>World Health Organization/European Medicines Agency<br>Journalists<br>Religious leaders<br>Friends and family<br>Government health authorities<br>Politicians | |
| 25 | Information sources: Exposure | Which sources of information are you most exposed to about COVID-19? Please rank from most to least exposed. | Likert | [Ranking question]<br>Local health workers<br>Scientists and health experts<br>World Health Organization/European Medicines Agency<br>Journalists<br>Religious leaders<br>Friends and family<br>Government health authorities<br>Politicians | |

| | | | | | |
|---|---|---|---|---|---|
| 26 | Information: Objective knowledge can encourage vaccine uptake | Which of the following aspects of COVID-19 information have you appreciated/do you appreciate the most? | Multiple choice (select all that apply) | Written in a clear, concise manner = 0<br>Written from a scientific point of view = 1<br>Makes me feel like I am the person they are speaking to = 2<br>Provides all the information that I need to make a good decision = 3<br>Is respectful of me and my choices=4 | |
| 27 | Information sources: Misinformation | Have you ever received or heard negative information about vaccinations? | Y/N | No =0<br>Yes =1 | |
| 28 | Information sources: Misinformation | Did hearing this negative information affect your decision to get a vaccine, or not get a vaccine? | Y/N | No =0<br>Yes =1 | |
| 29 | Participation in the FGD | Would you be interested in participating in a focus group discussion? | Y/N | No=0<br>Yes =1 | |
| | | Please provide your name, phone number, and email address | Open response | ____ | [For those who said yes to the question above] |
