# Supplementary material for: Using Behavior Integration to Identify Barriers and Motivators for COVID-19 Vaccination and Build a Vaccine Demand and Confidence Strategy in Southeastern Europe
Source: Vaccines (Basel). 2024 Oct 2;12(10):1131. doi: 10.3390/vaccines12101131 (PMC11511038; doi:10.3390/vaccines12101131)
Supplement: Supplementary file 1 [file vaccines-12-01131-s001.zip › Supplementary Material 5.pdf]

Supplementary Material 5. Formative Assessment Qualitative Results

The research identified several key barriers to COVID-19 vaccination. Concerns about vaccine safety, side effects, and efficacy were prevalent, particularly among vulnerable groups such as pregnant women, individuals with chronic diseases, and those intending to have children. Doubts about efficacy were exacerbated by cases of COVID-19 infections post-vaccination and limited understanding of how efficacy might vary by vaccine type and COVID-19 variant. Misconceptions about the rapid development and approval processes of vaccines also contributed to hesitancy. Another significant barrier was the importance placed on personal freedom and autonomy, especially among younger individuals who resisted perceived coercion to vaccinate. Complacency toward the pandemic, driven by a belief that COVID-19 no longer posed a significant threat, also discouraged vaccination. Deeply ingrained myths and misconceptions about adverse effects on reproductive health made people resistant to official messages. A pervasive lack of trust in government and media, perceived as politically motivated rather than health-focused, compounded these barriers. Social factors including widespread misinformation, lack of public counter-messaging, insufficient data transparency, and the absence of recommendations from health care providers, further undermined vaccine acceptance.

The research also identified several motivators for COVID-19 vaccination. Concern for personal health and fear of contracting the disease motivated people who were health-conscious or had chronic conditions like diabetes to get vaccinated. These individuals were often influenced by their health care providers' recommendations. Additionally, protecting vulnerable family members such as elderly relatives was a compelling reason for younger individuals to seek vaccination. Job and financial security emerged as practical motivators, with some health care workers, for instance, incentivized through salary bonuses or the threat of job reassignment. The desire to travel abroad, where vaccination was often a prerequisite, was a significant motivator, particularly for younger individuals.

Doctors' recommendations significantly shaped peoples' decisions. While some advised against vaccination for oncological, cardiovascular, and autoimmune patients, endocrinologists generally supported vaccination for diabetic patients, indicating a collective professional endorsement of its benefits. Peer influence among doctors also affected their stances, as some were reluctant to contradict colleagues' anti-vaccine positions. Family members and friends influenced vaccination decisions through personal vaccine experience, positive or negative. Religious leaders, particularly within the Orthodox Church, opposed vaccination or remained neutral, exerting a negative influence among their followers. Anti-vaccine activists, often appearing as credible and reasonable voices, spread misinformation. Scientists presented a mixed influence, with some promoting vaccination and earning public trust as others undermined it. The mass media predominantly amplified anti-vaccine narratives and focused on negative framing, such as emphasizing stories of the unvaccinated population, which fostered skepticism. The Serbian Medical Chamber and the Chamber of Nurses failed to lead public communication about vaccines, inadvertently giving rise to conspiracy theories.

Table 1. Key Influencers

Influencer	Description
**Doctors**	Doctor recommendation is very influential in people's vaccination decision.<br>● Recommendation NOT to get vaccinated common among oncological, cardiovascular, and autoimmune patients.<br>● Many diabetes patients have received the vaccine. It is possible that endocrinologists believe in the value of and recommended the vaccine to their patients.<br><br>Peer influence: Doctors who are pro-vaccine may be reluctant to contradict anti-vaccine colleagues.
**Family and friends**	Family members' and friends' experiences with COVID-19 vaccine (e.g., side effects) affect peoples' decisions.
**Church leaders**	Orthodox Church opposed to or silent about vaccination.
**Anti-vaccine activists**	Includes doctors who spread misinformation through social networks (and may be paid to do so).
**Scientists**	Some scientists promoted vaccination and were trusted by the population.
**Mass media**	Mass media communications amplified the voice of anti-vaccine activists/conspiracists and fostered vaccine skepticism.<br>● Mass-media communication gave too much visibility to those who spoke against vaccination.
**Government**	Government actors responsible for managing the pandemic (and the vaccine program) were viewed as passive and did not lead the COVID-19 response effectively. This gave room to vaccine conspiracists and anti-vaccine activists.

**Medical organizations**	The medical/science/public health community is not the main source of public information on COVID-19 vaccines (and neither is the government). This allowed conspiracy theories to flourish.<br><br>Organizations (e.g., Serbian Medical Chamber and Chamber of Nurses) did not publicly promote COVID-19 vaccine or counter misinformation.<br><br>Some doctors may have been rewarded financially to create confusion about the importance of vaccines (e.g., some promoted probiotics, while others said it was not important).
