# Supplementary material for: Using Behavior Integration to Identify Barriers and Motivators for COVID-19 Vaccination and Build a Vaccine Demand and Confidence Strategy in Southeastern Europe
Source: Vaccines (Basel). 2024 Oct 2;12(10):1131. doi: 10.3390/vaccines12101131 (PMC11511038; doi:10.3390/vaccines12101131)
Supplement: Supplementary file 1 [file vaccines-12-01131-s001.zip › Supplementary Material 6.pdf]

Supplementary Material 6. Formative Assessment Quantitative Results

Table 1. Barriers to Vaccination in Moldova and Serbia

**Barrier**	**Moldova**		**Serbia**	
	Urban	Rural	Urban	Rural
	OR (95% CI)	OR (95% CI)	OR (95% CI)	OR (95% CI)
**Safety concerns**	1.839 (1.328–2.547)**	1.145 (0.792–1.655)	1.990 (1.351–2.931)**	1.741 (1.071–2.831)*
**Could not get preferred vaccine**	2.803 (0.975–8.055)	1.531 (0.445–5.263)	1.170 (0.247–5.539)	1.257 (0.142–11.163)
**Difficulty making an appointment (e.g., technical issues)**	1.524 (0.444–5.236)	5.311 (0.855–32.976)	1.475 (0.302–7.199)	0.552 (0.069–4.444)
**Difficulty getting to vaccination site**	2.298 (0.501–10.544)	0.543 (0.063–4.646)	1.001 (0.302–1.198)	0.343 (0.069–1.05)
**Waiting times too long**	2.362 (0.788–7.080)	0.5691 (0.066–5.328)	3.222 (1.138–9.135)*	0.292 (0.258–3.348)
**Unable to leave work duties/ school/child care**	8.616 (2.603–28.523)**	1.697 (0.299–9.627)	0.571 (0.071–4.579)	6.455 (0.558–7.444)

* p < .05; ** p < .01

Table 2. Motivators of Vaccination in Moldova and Serbia

Motivator	Moldova		Serbia	
	Urban	Rural	Urban	Rural
	OR (95% CI)	OR (95% CI)	OR (95% CI)	OR (95% CI)
**Recommendation from health care provider**	0.533 (0.292–0.972)*	0.336 (0.162–0.696)**	0.859 (0.446–1.656)	0.480 (0.186–1.239)
**Pleasant interaction with health care provider**	0.817 (0.567–1.177)	0.712 (0.463–1.097)	0.568 (0.356–0.907)*	0.400 (0.213–0.751)**
**Understandable information**	0.578 (0.415–0.805)**	1.108 (0.743–1.651)	0.799 (0.527–1.213)	0.425 (0.246–0.736)**
**Collective responsibility**	0.926 (0.510–1.602)	0.844 (0.318–2.243)	0.627 (0.306–1.285)	0.939 (0.340–2.577)
**Vaccine efficacy**	0.711 (0.384–1.317)	1.029 (0.495-2.136)	0.755 (0.331–1.721)	0.325 (0.088–1.203)
**Descriptive norms—most adults vaccinated**	0.475 (0.236–0.955)*	0.462 (0.204–1.046)	0.313 (0.149–0.655)**	0.338 (0.127–0.896)*
**Proscriptive norms—community leaders**	2.276 (1.292–4.012)**	1.835 (0.899–3.743)	1.704 (0.850–3.414)	1.300 (0.482–3.509)
**Proscriptive norms—family members**	0.443 (0.250–0.785)**	0.463 (0.226–0.948)*	0.364 (0.169–0.784)*	0.639 (0.224–1.823)
**Proscriptive norms—religious leaders**	4.835 (1.318–17.667)*	0.10 (0.050–0.150)	1.042 (0.121–8.981)	0.478 (0.056–4.187)
**Trust in local health care providers**	0.719 (0.570–0.909)**	0.955 (0.700–1.302)	0.790 (0.617-1.010)	0.608 (0.428–0.873)**

**Trust in scientists and health experts**	0.850 (0.671-1.077)	0.957 (0.697–1.314)	0.880 (0.681–1.137)	0.699 (0.505–0.966)*
**Trust in journalists**	0.904 (0.721–1.134)	0.967 (0.732–1.729)	1.058 (0.825–1.356)	0.706 (0.496–1.007)

* p < .05; ** p < .01
