# Supplementary material for: Using Behavior Integration to Identify Barriers and Motivators for COVID-19 Vaccination and Build a Vaccine Demand and Confidence Strategy in Southeastern Europe
Source: Vaccines (Basel). 2024 Oct 2;12(10):1131. doi: 10.3390/vaccines12101131 (PMC11511038; doi:10.3390/vaccines12101131)
Supplement: Supplementary file 1 [file vaccines-12-01131-s001.zip › Supplementary Material 7.pdf]

Supplementary Material 7. Priority Behaviors, Behavior Profiles, and Pathways to Change

Moldova

1. As part of a healthy lifestyle, pregnant women of any age get full course of COVID-19 vaccine.
2. As part of a healthy lifestyle, people ages 45+ with a chronic health condition (e.g., hypertension, cardiopulmonary disease [COPD]) get full course of COVID-19 vaccine.
3. As part of their health care, Ukrainian refugees get full course of COVID-19 vaccine.
4. As part of a healthy lifestyle, medical specialists recommend full course of COVID-19 vaccines for pregnant women and/or patients with chronic health conditions when appropriate.

North Macedonia

1. As part of a healthy lifestyle, people ages 45+ with a chronic health condition (e.g., hypertension, COPD) get full course of COVID-19 vaccine.
2. As part of a healthy lifestyle, health professionals get full course of COVID-19 vaccine.
3. As part of a healthy lifestyle, medical specialists recommend full course of COVID-19 vaccines for pregnant women and/or patients with chronic health conditions when appropriate.
4. As part of a healthy lifestyle, family doctors provide full course of COVID-19 vaccines to pregnant women and/or patients with chronic health conditions when indicated as appropriate by patients' specialists.

Serbia

1. As part of a healthy lifestyle, pregnant women of any age get full course of COVID-19 vaccine.
2. As part of a healthy lifestyle, people ages 45+ with a chronic health condition (e.g., hypertension, COPD) get full course of COVID-19 vaccine.
3. As part of a healthy lifestyle, Serbian pregnant women with chronic health conditions (e.g., hypertension, COPD) get full course of COVID-19 vaccine.
4. As part of a healthy lifestyle, health professionals get full course of COVID-19 vaccine.
5. As part of a healthy lifestyle, medical specialists recommend full course of COVID-19 vaccine for pregnant women and/or patients with chronic health conditions when appropriate.
6. As part of a healthy lifestyle, primary care health professionals provide full course of COVID-19 vaccines to pregnant women and/or patients with chronic health conditions when indicated as appropriate by patients' specialists.
