# Supplementary material for: Using Behavior Integration to Identify Barriers and Motivators for COVID-19 Vaccination and Build a Vaccine Demand and Confidence Strategy in Southeastern Europe
Source: Vaccines (Basel). 2024 Oct 2;12(10):1131. doi: 10.3390/vaccines12101131 (PMC11511038; doi:10.3390/vaccines12101131)
Supplement: Supplementary file 1 [file vaccines-12-01131-s001.zip › Supplementary Material 8.pdf]

Supplementary Material 8. Factors and Strategies Based on the Project's Behavior Profiles

Table 1: Factors and Strategies based on the Project's Behavior Profiles

Factor	Strategy
**Enabling Environment**	
**Financing**	Work with the Health Insurance Fund to create a strategy to allocate additional funds for health promotion at the secondary and tertiary levels (medical specialists).
**Institutional capacity building**	Revise/improve Institute of Public Health (IPH) platform (e.g., develop site changes, input documents, maintain site, train staff to analyze and curate incoming information).<br><br>Build capacity of IPH and Ministry of Health (MOH) to provide high-quality health information to public and medical professionals.
**Partnerships and networks**	Create a package of all materials and activities (including BI) for learning exchanges with all countries (and when appropriate, develop capacity-building exercises).<br><br>Train pharmacists on healthy lifestyle behaviors through sessions focused on quality customer service.<br><br>Encourage UNICEF and WHO to share project information regionally, such as by holding conferences.
**Policies and governance**	Develop clear guidelines for medical professionals on healthy lifestyles at different points of the health continuum (e.g., pregnancy, chronic health conditions), including the importance of COVID-19 vaccine.
**Data-driven decision making**	Work with IPH and MOH to increase use of available data to foster learning exchange and comparison of vaccine coverage and other relevant information.
**Systems, Products, and Services**	
**Quality improvement - health professional skills**	Develop pre- and in-service sessions on healthy lifestyle, including the use of COVID-19 vaccine (coaching, peer mentoring) for medical specialists and health professionals (including how to properly communicate facts).<br><br>Conduct high-quality client service provision training including respectful care, counseling, and interpersonal communication for medical health professionals.<br><br>Train health professionals on the use of the IPH platform (understanding, feedback, etc.).

**Quality improvement - journalist skills**	Conduct training sessions with journalists (Internews, journalist faculty, etc.).
**Quality improvement - NGO skills**	Train nongovermental organization staff to conduct activities and use materials.
**Quality improvement - nurse skills**	Create opportunities for sharing and learning with health care workers (nurses) within their institute/health care facility.
**Quality improvement - pharmacist skills**	Train pharmacists on healthy lifestyle behaviors through sessions focused on quality customer service.
**Technology - health information system (HIS)**	Work with the MoH to add functionality to the HIS to remind specialists to discuss health lifestyle behaviors and indications (diagnostic codes) and contraindications for vaccines (flu and COVID-19) with high-risk patients.
**Technology - IPH platform**	Add information on healthy lifestyle with open access for the public and sign in for health professionals.
**Demand and Use**	
**Advocacy with stakeholders and health professionals**	Develop a series of evidence-based infographics (to show that preserving health and vaccinating save money) to help health professionals understand the value of COVID-19 vaccines as part of a healthy lifestyle.
**CE with health professionals**	Organize breakfasts with health professionals to discuss challenges and opportunities for healthy lifestyles including COVID-19 vaccine as part of a broader set of health lifestyle behaviors.
**CE with key populations**	Hold meetings at community centers to discuss information on healthy lifestyles and tactics for support.
**CE with major stakeholder**	Ask major stakeholders to review and provide feedback on materials developed for healthy lifestyles.
**Communication for health staff**	Develop (review and update) healthy lifestyle posters, radio spots, SMS messages, fact sheets, and/or flyers for medical professionals.
**Communication for key populations**	Develop a series of interactive exercises that cover healthy lifestyles with a focus on COVID-19 vaccine and include games, role plays, discussion scenarios, posters, flyers, and handouts on the following topics: vaccination in general and specific to their location (e.g., online search tool, map); available vaccines; and procedures (e.g., contact info, responsible person).
**Communication with key populations**	Consider "behaviorally focused" motivational interviewing.
**Skills building for key populations**	Organize sessions on how to communicate, for example with a provider.

Table 2: Supporting Actors

Institute of Public Health
Ministry of Health
Pharmaceutical regulatory agency
Professional chambers
Health professional associations
Patient organizations
Civil society and community-based organizations
Media/journalists
