# Supplementary material for: Using Behavior Integration to Identify Barriers and Motivators for COVID-19 Vaccination and Build a Vaccine Demand and Confidence Strategy in Southeastern Europe
Source: Vaccines (Basel). 2024 Oct 2;12(10):1131. doi: 10.3390/vaccines12101131 (PMC11511038; doi:10.3390/vaccines12101131)
Supplement: Supplementary file 1 [file vaccines-12-01131-s001.zip › Supplementary Material 9.pdf]

Supplementary Material 9. CE Workshop Participant In-depth Interview Guide

Potential Audiences (TBD): Vaccinated, unvaccinated CE workshop participants

Purpose:

To explore the experiences of both vaccinated and unvaccinated CE participants in accessing COVID-19 vaccines and adhering to the commitments made during the workshop.

Ideally, we'd like to interview ~2 vaccinated, and ~2 unvaccinated individuals per country (as feasible)

Discussion Preparation:

1. Reconfirm that each participant matches the criteria set for participation above

2. Work with Association Duga/ESE/PAS Center to identify participants

3. Ensure that each participant has signed the participation form

Introduction:

My name is _______, who will facilitate this discussion, and this is _______, our notetaker for today. We are here to discuss and receive your feedback on the CE event you participated in and your experiences in accessing COVID-19 vaccines or adhering to the commitments you made during the CE event. All responses today are confidential and your names or any personal information will not be associated with this information in any way later.

There are no right or wrong answers, just your opinions and thoughts on COVID-19 vaccinations. This information will be used to both help us adapt our activities and materials to assist in COVID-19 efforts. We would like to tape the session so that we can listen to it again afterwards to ensure that we captured your ideas and thoughts accurately.

We greatly appreciate you agreeing to participate in this discussion and are so happy to have you here. Let's begin. Our conversation should take no more than 30 minutes.

Overview Questions:

1. Thank you for attending our workshop. How was your overall experience attending the workshop?

Probes:

- *Which topics (e.g, health lifestyles, vaccines) did you find most useful?*
- *Which interactive activities/games did you find helpful in understanding the content?*
- *What aspects of the workshop do you think can be improved?*
- *Would you recommend this workshop to other peers?*

2. Now that you have attended the workshop, how has your perception about COVID-19 evolved? Can you share your thoughts and feelings about COVID-19 in general?

3. What commitments or actions did you make at the end of the CE event? Why did you select these ones?

4. Which commitments were you able to follow through with? What factors helped you in keeping the commitments you made?

5. Which commitments were you not able to follow through? What factors have hindered you from keeping the commitments you made? Probe for issues with accessing vaccine and barriers to getting an appointment or referral.

6. [For those that got vaccinated]: Can you take me through the step by step process you took to get vaccinated?

Probes: Where did you get vaccinated? What was your experience with the quality of vaccination service you received? What were your interactions like with the vaccinator/health provider?

7. [For those that did not get vaccinated]: Which commitments are you planning to follow through with? What additional support do you need to help you keep the commitments you made?

Closing:

Thank you for taking time out of your day to participate and share ideas and opinions with us. We are so thankful to you.
